# Biomarker for early renal microvascular and diabetic kidney diseases

**DOI:** 10.1080/0886022X.2017.1323647

**Published:** 2017-05-11

**Authors:** Narisa Futrakul, Prasit Futrakul

**Affiliations:** a Department of Physiology, Faculty of Medicine, King Chulalongkorn Memorial Hospital, Chulalongkorn University, Bangkok, Thailand;; b Academy of Science, The Royal Institute of Thailand and Bhumirajanagarindra Kidney Institute, Bangkok, Thailand

**Keywords:** Diabetic kidney disease, endostatin, renal microvascular disease, renal hemodynamics, FE Mg

## Abstract

Recognition of early stage of diabetic kidney disease, under common practice using biomarkers, namely microalbuminuria, serum creatinine level above 1 mg/dL and accepted definition of diabetic kidney disease associated with creatinine clearance value below 60 mL/min/1.73 m^2^, is unlikely. This would lead to delay treatment associated with therapeutic resistance to vasodilator due to a defective vascular homoeostasis. Other alternative biomarkers related to the state of microalbuminuria is not sensitive to screen for early diabetic kidney disease (stages I, II). In this regard, a better diagnostic markers to serve for this purpose are creatinine clearance, fractional excretion of magnesium (FE Mg), cystatin C. Recently, renal microvascular disease and renal ischemia have been demonstrated to correlate indirectly with the development of diabetic kidney disease and its function. Among these are angiogenic and anti-angiogenic factors, namely VEGF, VEGF receptors, angiopoietins and endostatin. With respect to therapeutic prevention, implementation of treatment at early stage of diabetic and nondiabetic kidney disease is able to restore renal perfusion and function.

## Introduction

It has been a general acceptance that the therapeutic outcome of diabetic kidney disease under current common practice is rather unsatisfactory in preventing the progressive increment in number of patients entering end-stage renal disease requiring renal replacement therapy [1]. The crucial issue responsible for this is rather due to the inappropriate diagnostic markers available such as microalbuminuria, the level of serum creatinine above 1 mg/dL, the accepted recognition of diabetic kidney disease or chronic kidney disease, only when the creatinine clearance is below 60 mL/min/1.73 m^2^ [2–6]. Such conceptual view leads to the late implementation of treatment at a rather late stage (stage 3). Treatment at this stage is usually unable to restore renal function but simply slows the renal progression towards end-stage renal disease. Such practice reflects the fact that most of the early stage of diabetic kidney disease patients are widely ignored and under recognized.

**Figure 1.**
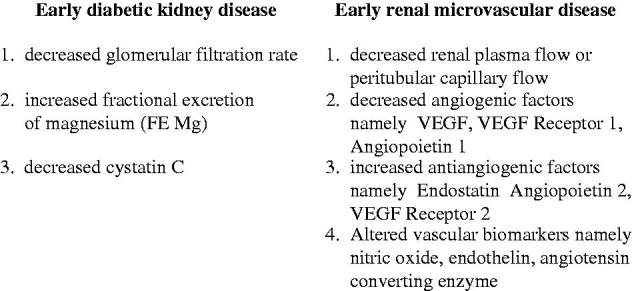
Indices indicating early diabetic kidney disease and renal microvascular disease.

To overcome the above clinical pitfall and hopefully to improve the outcome of diabetic kidney disease, a better diagnostic marker enable to identify the early stage of diabetic kidney disease would address to this issue. In this regard, a determination of creatinine-based GFR estimates is better than serum creatinine level per se but is limited in hyperfiltration status [7,8]. A variety of alternative biomarkers such as cystatin C is shown to correlate with GFR [9,10] especially at the early stage of diabetic kidney disease [11–13]. Tubular biomarkers such as neutrophil gelatinase-associated lipocalin (NGAL), kidney injury molecule 1 (K1M1) N-acetyl-B-(D) glucosaminidase (NAG), advanced glycation end products (AGES), namely pentosidine had been related to the status of microalbuminuria and appeared to be not a proper index for the early detection of diabetic kidney disease due to its relationship with the insensitive marker microalbuminuria [14–18]. Recently, a tubular biomarker fractional excretion of magnesium (FE Mg) releasing from the breakdown of high-energy ATP (magnesium attached) during the tubular cell damage has been shown to correlate directly with the magnitude of tubulointerstitial disease at the early onset of chronic kidney disease. In clinical renal disease associated with an intact tubulointerstitium such as acute poststreptococcal glomerulonephritis or minimal-change steroid-sensitive nephrosis, the value of FE Mg is usually within normal limit as compared to the healthy subject. In contrast, FE Mg becomes abnormally elevated and correlates with the degree of tubulointerstitial disease in non-diabetic chronic kidney disease [19,20]. In this regard, FE Mg has also been shown to correlate with the clinical severity of diabetic kidney disease. In mild and early stage of diabetic kidney disease during the stage of normoalbuminuria, the mean values of FE Mg were 4.1 ± 1%, whereas the mean values of FE Mg were increased to 6.6 ± 2% in albuminuric stage of type-2 diabetes mellitus. Furthermore, FE Mg is also demonstrated to inversely correlate with the degree of reduction in peritubular capillary flow [21,22]. Thus, the diagnostic biomarkers that are able to recognize early diabetic kidney disease include the decreased (e) creatinine clearance, or glomerular filtration rate, the increased fractional excretion of magnesium (FE Mg) and the decreased cystatin C (Figure 1). However, the diagnosis of early stage of diabetic kidney disease would be interpreted with caution in some patients during the state of hyperfiltration, since it may lead to overestimation.

## Subclinical manifestation of renal microvascular disease in chronic kidney disease and diabetic kidney disease

Renal microvascular disease has been reiterated to correlate with the clinical severity of a variety of non-diabetic and diabetic chronic kidney diseases. Bohle and associates noted an inverse correlation between the post-glomerular capillary patency and the development of tubulointerstitial filbrosis [23,24]. Futrakul et al. also demonstrated that reduced endothelial factor VIII staining in renal microcirculation correlates with haemodynamic alteration in nephrosis. In this regard, a greater loss of endothelial cell in renal microcirculation was observed in severe form of nephrosis (focal segmental glomerulosclerosis) than that documented in mesangial proliferative nephrosis. Such finding of renal microvascular disease concurred with the reduction in renal plasma flow and peritubular capillary flow that was greater in nephrosis associated with focal segmental nephrosis and lesser in mesangial proliferative nephrosis [25]. In addition, the reduction in renal plasma flow is usually preceded the development of tubulointerstitial fibrosis [26].

With respect to the diabetic kidney disease, such correlation has been sporadically reported due to the limitation of histopathologic study. In type-2 diabetic kidney disease, there is a rather persistent pattern of reduction in renal perfusion implying renal microvascular disease. Recently, it is shown that a reduction in renal plasma flow and peritubular capillary flow has been demonstrated in early stage of diabetic kidney disease (stages 1,2) during the stage of normoalbuminuria [27–30]. Evidence of diabetic kidney disease is relevant to the reduction in creatinine clearance or glomerular filtration rate. In addition, evidence of tubulointerstitial disease is also implied by the abnormal elevation of fractional excretion of magnesium (FE Mg).

**Figure 2.**
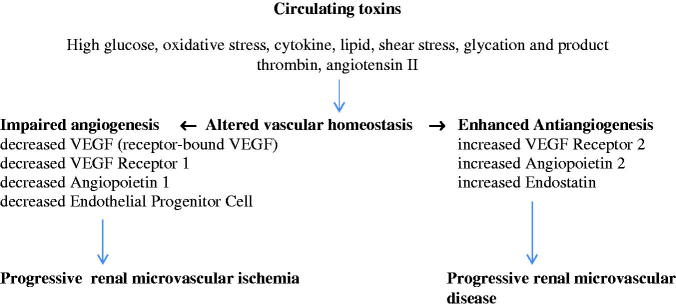
Altered vascular homoeostasis in diabetic renal microvascular disease.

The pathogenetic mechanism of diabetic renal microvascular disease is triggered by various circulating toxins, namely high glucose [31,32], oxidation stress [33], cytokine [34,35], lipid, shear stress [36], thrombin and angiotensin II [37]. Such circulating toxins induce endothelial cell injury by which it detaches endothelial cell, VEGF receptor bound into the circulation [38] as well as induces endothelial cell apoptosis [39] (Figure 2). Injury to endothelial cell alters the cell surface to become procoagulant, provasoconstrictive and express adhesion molecule. In addition, impaired angiogenic factors such as receptor-bound VEGF, a decreased VEGF receptor 1, a decreased angiopoietin 1 and at late stage a decreased endothelial progenitor cell have been demonstrated, which would impair the process of angiogenesis. Taken together, all these factors would induce a progressive renal microvascular ischemia. Moreover, an enhanced antiangiogenesis has also taken place which includes an increased VEGF receptor 2, an increased angiopoietin 2 and an increased endostatin [40–53]. These antiangiogenic factors would induce a progressive damage to the vascular wall, namely the apoptosis of the endothelial cells, the proliferation of vascular smooth muscle cell and the progressive renal microvascular disease. Such altered vascular homoeostasis would explain the therapeutic resistance to vasodilator treatment as well as the progressive decline in renal perfusion observed in late state of diabetic kidney disease.

With respect to the diagnostic markers of early stage of renal microvascular disease, the renal hemodynamic study namely renal plasma flow, peritubular capillary flow (renal plasma flow minus glomerular filtration rate) would be the direct tool to reflect the early stage of renal microvascular disease. Indirectly, the study of vascular homeostasis such as angiogenic factors namely VEGF, VEGF receptor 1, angiopoietin 1, as well as antiangiogenic factors, namely angiopoietin 2, VEGF receptor 2 and endostatin would also reflect the early stage of renal microvascular disease. Furthermore, recent study on vascular biomarkers, namely nitric oxide, endothelin, angiotensin converting enzyme in normotensive, normoalbuminuric, normal or increased creatinine clearance in patients associated with type-2 diabetes, has revealed a significant alteration indicating an abnormal vascular status or renal microvascular disease in this early stage of diabetic kidney disease [54,55]. Further support to this conceptual view, Fernand Mae-Moune Lai and associated had reported an isolate diffuse thickening of glomerular basement membrane indicating an early renal microvascular disease in prediabetes [56]. Wacharasindhu and associates also showed a significant reduction in peritubular capillary flow and a high value of glomerular filtration rate in the presence of reduced renal perfusion characteristic of glomerular hyperfiltration in both type of early stage of childhood diabetes [57].

## Endostatin: a biomarker of renal microvascular disease

**Figure 3.**
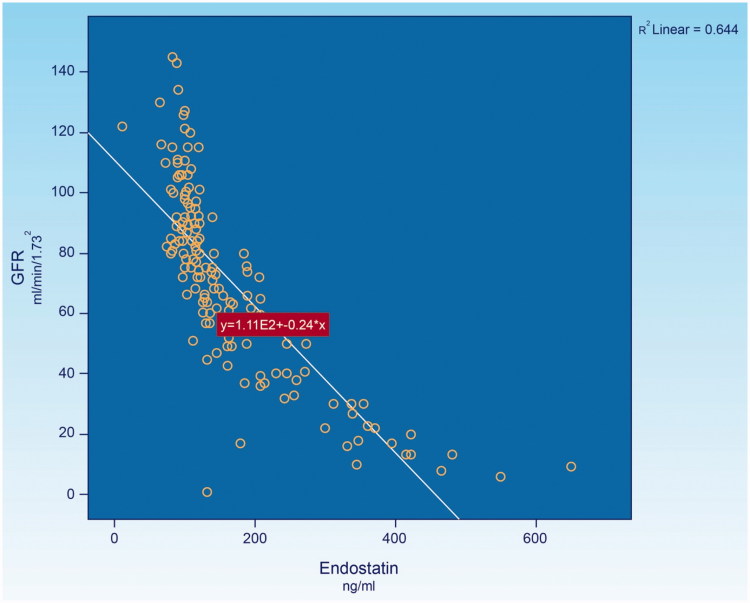
Demonstrates the relationship between endostatin and GFR in diabetic kidney disease.

Endostatin, a 20 kDa (terminal fragment of collagen XVIII) is a potent endothelial cell-specific inhibitor of angiogenesis reported in several experimental studies [58–60]. Recently, experimental studies also suggested that endostatin might lead to the rarefaction of renal microvasculature. Endostatin is a broad-spectrum angiogenesis inhibitor and may interfere with the pro-angiogenic action of growth factors such as basic fibroblast growth factor and vascular endothelial growth factor. *In vitro* studies, it has been shown that endostatin blocks the proliferation and organization of endothelial cells into new blood vessel [61]. In animal studies, endostatin inhibited angiogenesis and growth of both normal tissue and cancerous tissue [60]. Endostatin represses cell-cycle control and antiapoptosis genes in proliferating endothelial cells, resulting in cell death [62,63]. An abnormally elevated plasma levels of endostatin was encountered in chronic kidney disease [64,65]. However, the correlation between the levels of endostatin and the stages of chronic kidney disease has not as yet been established. In this regard, recent study had assessed of such correlation in 160 chronic kidney disease patients associated with various degrees of renal functional impairment. The result indicated that there was a significantly reversal correlation between the levels of endostatin and the level of glomerular filtration rate; (Figure 3), the lower the glomerular filtration rate, the higher the level of endostatin. Since glomerular filtration rate has been noted to correlate directly with the level of renal plasma flow [66], the elevated level of endostatin in the presence of low level of glomerular filtration rate or the reduced renal plasma flow would indirectly reflect the degree of renal microvascular disease. Recently, it was noted that the level of endostatin is significantly elevated even at the early stage of diabetic kidney disease associated with normoalbuminuria, normal or increased creatinine clearance in type-2 diabetes [67]. This would imply that renal microvascular disease is likely to develop at the very early stage of chronic kidney disease or diabetic kidney disease.

## When would be the optimal time to prevent the development of renal microvascular disease?

Under common practice attached to the concept of accepting microalbuminuria, or the level of serum creatinine above 1 mg/dL as the diagnostic index. The early stage of renal microvascular disease inducing early impact on the development of diabetic kidney disease has usually been under recognized. The above diagnostic markers usually miss the early stages (I, II) of diabetic kidney disease. Implementation of the treatment of diabetic kidney disease is usually initiated at a rather late stage. Vascular response to vasodilator treatment in microalbuminuric diabetic kidney disease failed to correct the status of renal ischemia but simply slowed the renal disease progression. This is in accordance with the intrarenal hemodynamic study during pre-treatment and post-treatment period with vasodilators containing ACEI Enarapril 10–20 mg/d, ARB Telmisartan 40–80 mg/d ± calcium channel blocker in 29 microalbuminuric diabetic kidney disease patients. Following vasodilator treatment, progressive reductions in renal plasma flow and glomerular filtration rate, as well as a progressive increase in both afferent and efferent renal arteriolar resistance were also noted [68].

To overcome the present preventive and therapeutic strategic failure under common practice, an alternative therapeutic approach has been launched to implement an early preventive strategy at the early stage of diabetic kidney disease (diabetic kidney disease stage I, II). Such conceptual view has been supported by the study of vascular homoeostasis in early stage of both non-diabetic and diabetic kidney disease reveals a normal or mild impairment. Recent study in 50 patients associated with normoalbuminuric diabetic kidney disease stage II had revealed significant increases in renal plasma flow (pre-treatment 470 ± 57 versus post-treatment 530 ± 45 mL/min/1.73 m^2^), peritubular capillary flow (pre-treatment 382 ± 62 vs. post-treatment 433 ± 485 mL/min/1.73 m^2^) and glomerular filtration rate (pre-treatment 85 ± 25 vs. post-treatment 106 ± 30 mL/min/1.73 m^2^), following treatment with multidrug vasodilators, namely ACE inhibitor, angiotensin receptor blocker, ±calcium channel blocker in conjunction with correction of metabolic disorders, exercise, diet control for 24–36 months [69–71]. Similar therapeutic approach in 65 non-diabetic chronic kidney disease patients had also demonstrated a successful restoration of renal perfusion and glomerular filtration rate [72]. These findings imply that an adequate vascular repair is vulnerable to renal regeneration in early stage of both diabetic and non-diabetic kidney diseases.

With respect to the preceding successful achievement in early implementation of renal perfusion and function, The Bhumirajanagarindra Kidney Institute, the only Institute in the country to specially serve for this purpose has set up a community-based study to early screen and therapeutic intervention of diabetic and non-diabetic chronic kidney patients in the whole Province of Kampangpetch with satisfactory result in progress. Currently, the Ministry of Public Health of Thailand has already launched the above conceptual view of early screening and implementing early preventive strategy to cover 797 governmental hospitals throughout the country in 2016. The primary goal of this innovative strategic approach is to successfully minimize the progression toward end-stage renal disease, as well as to decline the utilization of renal replacement therapy in the near future.
